# Association of the Circulating Supar Levels with Inflammation, Fibrinolysis, and Outcome in Severe Burn Patients

**DOI:** 10.1097/SHK.0000000000001806

**Published:** 2021-05-20

**Authors:** Jian-Chang Lin, Xiao-Dong Chen, Zhao-Rong Xu, Lin-Wen Zheng, Zhao-Hong Chen

**Affiliations:** Fujian Provincial Key Laboratory of Burn and Trauma, Fujian Burn Institute, Fujian Burn Medical Center, Fujian Medical University Union Hospital, Fuzhou, Fujian, China

**Keywords:** Burn, hyperfibrinolysis, IL-10, pro/anti-inflammatory imbalance, suPAR, ABSI score, abbreviated burn severity index score, APTT, activated partial prothrombin time, AUC, area under the curve, CRP, C-reactive protein, IFN-γ, interferon γ, IL, interleukin, INR, international standardized ratio, ns, no significance, PAI-1, plasminogen activator inhibitor-1, PF-4, platelet factor 4, PT, prothrombin time, ROC, receiver operating characteristic curve, SOFA, sequential organ failure assessment, sP-selectin, soluble P-selectin, suPAR, soluble urokinase-type plasminogen activator receptor, TBSA, total burn surface area, TNF-α, tumor necrosis factor α, TT, thrombin time, uPA, urokinase-type plasminogen activator

## Abstract

**Background:**

Hyperfibrinolysis and pro/anti-inflammatory imbalance usually occur in the early stage of severe burns. Soluble urokinase-type plasminogen activator receptor (suPAR) is involved in fibrinolysis and inflammation. To date, the levels of circulating suPAR in non-survivors with severe burns remain unknown. This study aimed to investigate the early association between circulating suPAR levels and biomarkers of fibrinolysis, pro/anti-inflammatory, and prognosis.

**Methods:**

Sixty-four consecutive Chinese patients with severe burns and 26 healthy volunteers were enrolled in a prospective observational cohort. Clinical characteristics and laboratory data were collected prospectively. Blood samples were collected at 48 h post-burn, and suPAR and biomarkers of pro/anti-inflammatory and fibrinolysis were detected by enzyme-linked immunosorbent assays. Important indicators between non-survivors and survivors were compared. Linear regression analysis was performed to screen variables associated with suPAR. Logistic regression analysis and receiver operating characteristic curve (ROC) analysis were performed to evaluate the prognostic value of suPAR.

**Result:**

Compared with the control group, the circulating suPAR levels in the survivors (*P* < 0.001) and non-survivors (*P* = 0.017) were higher. Compared with survivors, non-survivors had lower circulating suPAR levels at 48 h post-burn, and they showed a higher degree of fibrinolysis (higher D-dimer) and a lower TNF-α/IL-10 ratio. According to linear regression analysis, the variables independently associated with a lower suPAR level were lower platelet factor 4 (PF-4), urokinase-type plasminogen activator (uPA), and TNF-α/IL-10 levels and a higher D-dimer level. Logistic regression and ROC analyses indicated that a suPAR level ≤ 4.70 μg/L was independently associated with 30-day mortality.

**Conclusion:**

Low circulating suPAR levels at 48 h post-burn in severe burn patients may reflect decreased TNF-α/IL-10 ratio and increased hyperfibrinolysis. suPAR can predict 30-day mortality in patients with severe burn.

## INTRODUCTION

Secondary hyperfibrinolysis and pro/anti-inflammatory imbalance usually occur in the early stage of severe burns (1, 2), and both are closely related to the poor prognosis of severe burn patients (3, 4). The cytokine network formed by inflammatory cytokines (IL-6, IL-8, and MCP-1) and anti-inflammatory cytokine (IL-10) may play a crucial role in the early hospital phase of major burn injury (2, 5). A study on the time course of pro-inflammatory and anti-inflammatory cytokine levels in patients with burns showed that early excessive anti-inflammatory (significantly elevated IL-10) has a poor prognosis in severe burn patients (3). However, none of these studies had data on the extent of pro-inflammatory and anti-inflammatory imbalance in the early stage of severe burns. Studies (6, 7) have shown that fibrinolysis activation may be related to systemic inflammatory reactions after burn injury, and it should be promising and meaningful to identify the link between them.

Urokinase-type plasminogen activator receptor (uPAR) is a single-chain glycoprotein that binds to the cell membrane surface by glycosylphosphatidylinositol at the C-terminus (8). The urokinase receptor system is a key regulator of the intersection among inflammation, coagulation, and fibrinolysis (9, 10). uPAR and its ligand urokinase plasminogen activator (uPA) provide a cell surface-integrated multimolecular complex that exerts pleiotropic functions influencing the development of inflammatory, immune, coagulation, and fibrinolytic responses (10, 11). uPAR is primarily expressed in immune cells and is cleaved from the cell surface through inflammatory stimulation to form soluble uPAR (suPAR) (12). suPAR could mediate similar biological functions by competing with uPAR for its ligand (13). We found (13, 14) that the concentration of suPAR depends on the activation level of the immune system and that suPAR is a stable marker of immune activation and cellular inflammation. Indeed, suPAR has been widely used as a prognostic marker for various diseases (15, 16).

Although suPAR has been widely investigated, few studies have been conducted in burn ICUs. Backes et al. (17) investigated the levels of alveolar lavage fluid and systemic suPAR in 11 patients with severe burn with inhalation trauma in 2011. Their findings were as follows: the pulmonary suPAR level was elevated in burn patients with inhalation trauma, and it correlated with pulmonary inflammation and coagulation; the systemic suPAR level correlated positively with the duration of mechanical ventilation and was a significant predictor of the duration of mechanical ventilation. However, they did not investigate the systemic suPAR levels in non-survivors with severe burns. In this study, we measured the circulating suPAR levels in 64 severely burned patients (including 11 non-survivors) at 48 h post-burn and then investigated the association between the circulating suPAR levels and biomarkers of fibrinolysis, pro/anti-inflammatory, and prognosis.

## PATIENTS AND METHODS

### Study design

This prospective observational cohort study was conducted at a burn medical center in China (Fujian Burn Medical Center).

### Ethical approval

This study was approved by the ethics committee of Fujian Medical University Union Hospital, and informed consent was obtained from each participant or their family representatives.

### Patients

The inclusion criteria were as follows: thermal burn patients aged 18 to 60 years; total burn surface area (TBSA) ≧ 30%; burn shock resuscitation ≦ 4 h after injury; hospitalized ≦ 12 h after injury. Patients with liver and renal insufficiency, a malignant tumor, rheumatic immune disease, or hematological diseases that affect the coagulation system were excluded retrospectively. Additionally, patients with craniocerebral trauma, visceral injury, or other severe combined injuries were excluded. The enrolled patients did not participate in any intervention trials. Patients could withdraw from the study at any time. Severe burn patients were divided into a non-survival group and a survival group according to the 30-day prognosis. Additionally, we recruited 26 healthy volunteers aged 18 to 60 years as the control group. The treatment plan of all the patients was consistent. During the burn shock period (0–48 h), no patient received anticoagulant/thrombolytic therapy or major surgery (e.g., escharotomy and skin grafting).

### Specimen collection and processing

The remaining arterial and venous blood samples of patients at 48 ± 2 h post-burn and remaining venous blood samples of healthy volunteers after physical examination were collected. Plasma (serum) samples were separated by centrifugation (Eppendorf Company, Germany) and stored in a freezer (Sanyo Company, Japan) at −80°C until the assays were performed.

### Data collection

The data on the following demographic and clinical parameters were collected from each patient: age, sex, TBSA, percentage of full-thickness burns, abbreviated burn severity index (ABSI) score (18), presence of inhalation injury, length of stay, sepsis complications, mechanical ventilation ratio, plasma supplementation (0–48 h), albumin supplementation (0–48 h), resuscitation fluid (0–48 h), and sequential organ failure assessment (SOFA) score (48 h).

### Assays

The numbers of monocytes, platelets, neutrophils, and lymphocytes, and the C-reactive protein (CRP) levels were measured using routine blood tests (CD600; Mindray, China). The pH and lactate levels were measured immediately after collecting the arterial blood samples by standard arterial blood gas analysis (ABL800; Redu, Denmark). The fibrinogen and D-dimer levels were measured by coagulation analysis (SEKISUI MEDICAL CO, Ltd, Japan).

The soluble biomarkers of pro/anti-inflammation, fibrinolysis, and platelet activation were detected by enzyme-linked immunosorbent assay (ELISA) according to the manufacturer's instructions (Shanghai Westang Bio-Tech Co, Ltd, China). The serum test items were platelet factor 4 (PF-4; ABC-ELISA; lower detection limit [LDL]: 0.2 ng/mL), interleukin 1β (IL-1β; ABC-ELISA; LDL: 1 pg/mL), interleukin 6 (IL-6; ABC-ELISA; LDL: 2 pg/mL), interleukin 4 (IL-4; ABC-ELISA; LDL: 1 pg/mL), interleukin 10 (IL-10; ABC-ELISA; LDL: 1 pg/mL), interleukin 13 (IL-13; ABC-ELISA; LDL: 3 pg/mL), interleukin 8 (IL-8; ABC-ELISA; LDL: 3 pg/mL), and interferon γ (IFN-γ; ABC-ELISA; LDL: 3 pg/mL). The citrate plasma test items included plasminogen activator inhibitor-1 (PAI-1; ABC-ELISA; LDL: 0.2 ng/mL). The EDTA plasma test items were soluble P-selectin (sP-selectin; ABC-ELISA; LDL: 60 pg/mL), uPA (ABC-ELISA; LDL: 16 pg/mL), tumor necrosis factor (TNF-α; ABC-ELISA; LDL: 15 pg/mL), and suPAR (ABC-ELISA; LDL: 16 pg/mL).

### Statistical analysis

Statistical analysis was performed using SPSS 21.0 (SPSS Inc, Chicago, Ill). Values are presented as means ± SD or % (n). *P* < 0.05 was considered statistically significant. The non-parametric Mann–Whitney test or Fisher exact test was performed to estimate the difference between groups. Non-parametric Spearman's correlation was performed to analyze the correlation between suPAR and continuous variables. The independent variables associated with suPAR were estimated using univariate and multivariate linear regression models. Univariate and multivariate logistic regression analyses were performed to estimate the independent predictors of the 30-day prognosis. Receiver operating characteristic (ROC) curves and areas under the curve (AUCs) were generated to compare the ability of suPAR and classical clinical biomarkers to distinguish the 30-day mortality.

## RESULTS

### Baseline characteristics of the study population

**Fig. 1:**
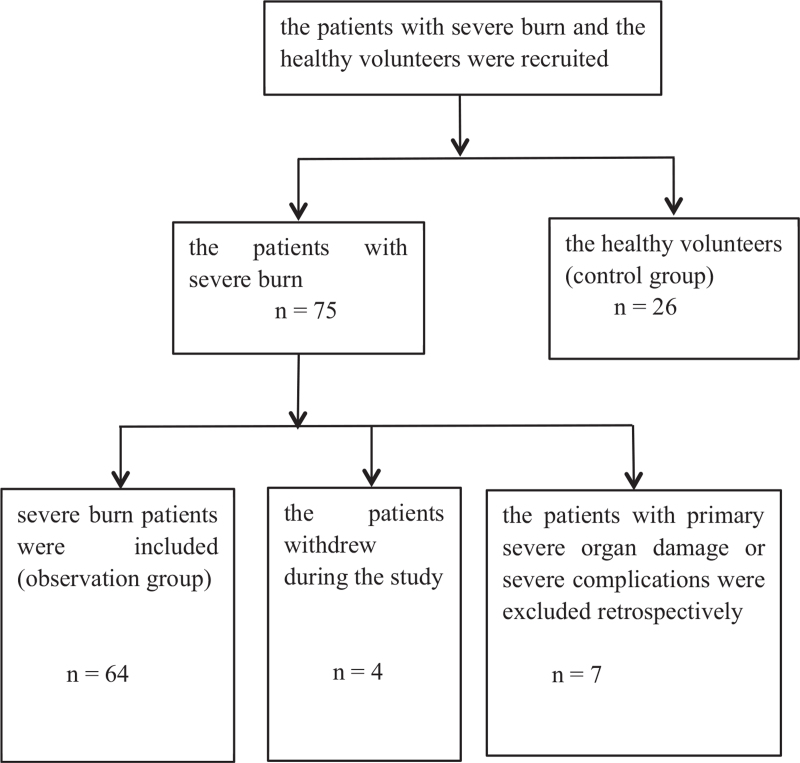
CONSORT diagram describing enrolment in the study.

A total of 75 patients met the inclusion criteria. Three patients with primary severe organ damage and four patients with severe complications were excluded retrospectively. Besides, four patients withdrew during the study. Therefore, 64 consecutive patients (79.7% men; mean age, 44.2 ± 10.5 years) and 26 healthy volunteers (80.8% men; mean age, 41.9 ± 10.6 years) were eligible for enrolment in the study (Fig. 1). Detailed information on the demographic and clinical characteristics is shown in Table 1. No significant differences were found in sex or age between the non-survival group and survival group or control group. On admission, the TBSA, percentage of full-thickness burns, ABSI score, and presence of inhalation injury in the non-survival group were significantly higher than those in the survival group. The SOFA score 48 h post-burn in the non-survival group was significantly higher than that in the survival group. Fifty-two of 64 severe burn patients (81.3%) were admitted to the burn ICU. The average survival time of the non-survivors was 19.9 ± 6.7 days. Twenty-three of 53 (43.3%) survivors and 10 of 11 (90.9%) non-survivors developed septic complications. The mechanical ventilation rate of the non-survivors was significantly higher than that of the survivors. In the burn shock stage, no additional platelet supplementation was provided to the non-survivors and survivors, but albumin supplementation and plasma supplementation in the non-survivors occurred at a significantly higher rate than in the survivors. The use of resuscitation fluid in the non-survivors was significantly greater than that in the survivors (Table 1).

**Table 1:** Clinical characteristics and laboratory parameters in non-survival and survival group at 48 h post-burn

		Non-survival group	Survival group	Control group	^∗^*P* value
Demographic and clinical characteristics					
N		11	53	26	
Gender	m (%)	72.7% (8)	81.1% (43)	80.8% (21)	ns
Age	Years	47.0 ± 8.1	43.5 ± 10.9	41.9 ± 10.6	ns
TBSA	%	83.6 ± 15.3	61.5 ± 20.0	\	0.001
Percentage of full-thickness burns	%	80.6 ± 15.3	26.7 ± 20.6	\	< 0.001
ABSI score	Score	12.6 ± 1.6	9.5 ± 2.4	\	< 0.001
SOFA score	Score	11.5 ± 2.0	3.2 ± 1.7	\	< 0.001
Presence of inhalation injury	% (n)	81.8% (9)	20.8% (11)	\	< 0.001
Admission to burn ICU	% (n)	100% (11)	77.4% (41)	\	ns
Length of stay	Day	19.9 ± 6.7	42.3 ± 21.0	\	< 0.001
Sepsis complications	% (n)	90.9% (10)	43.4% (23)	\	< 0.001
Mechanical ventilation ratio	% (n)	81.8% (9)	28.3% (15)	\	0.001
Plasma supplement (0–48 h)	ml/kg	67.8 ± 25.0	40.2 ± 22.0	\	0.005
Albumin supplement (0–48 h)	g/kg	1.50 ± 1.04	0.59 ± 0.38	\	0.017
Resuscitation fluid (0–48 h)	mL/kg•%TBSA	5.13 ± 1.03	4.23 ± 0.98	\	0.019
Blood cells and biochemical parameters					
Neutrophils	10^9^/L	16.8 ± 9.7	9.6 ± 5.9	3.0 ± 0.6	0.001
Lymphocytes	10^9^/L	1.15 ± 0.67	0.99 ± 0.36	1.80 ± 0.46	ns
Monocytes	10^9^/L	0.88 ± 0.53	1.04 ± 0.53	0.28 ± 0.07	ns
Platelets	10^9^/L	59.8 ± 23.5	104.6 ± 55.8	245.1 ± 30.6	0.011
pH	U	7.375 ± 0.124	7.377 ± 0.060	\	ns
Lactate	mmol/L	5.81 ± 2.54	4.39 ± 1.75	\	0.027
Fibrinolysis					
Fibrinogen	g/L	2.85 ± 1.10	4.40 ± 1.57	3.31 ± 0.78	0.003
D-dimer	mg/L	9.31 ± 6.87	2.41 ± 1.56	0.17 ± 0.14	0.008
PAI-1	μg/L	3.36 ± 3.04	3.30 ± 4.00	1.72 ± 1.45	ns
uPA	μg/L	1.46 ± 0.40	1.62 ± 0.78	2.80 ± 0.67	ns
suPAR	μg/L	3.42 ± 0.98	7.14 ± 3.87	2.49 ± 1.02	< 0.001
Platelet activation					
PF-4	mg/L	21.1 ± 8.0	27.5 ± 2.9	29.0 ± 2.2	0.025
sP-selectin	μg/L	125.6 ± 71.4	124.4 ± 54.3	67.0 ± 27.2	ns
Pro-inflammatory and anti-inflammatory					
CRP	mg/L	44.7 ± 33.6	110.2 ± 68.5	10.5 ± 5.6	< 0.001
IL-4	ng/L	110.0 ± 91.4	71.0 ± 83.4	24.7 ± 45.3	ns
IL-13	ng/L	127.3 ± 111.0	84.6 ± 103.6	30.5 ± 52.3	ns
IL-10	ng/L	85.0 ± 43.7	24.8 ± 22.5	3.3 ± 2.6	0.001
IL-1β	ng/L	24.5 ± 20.1	20.6 ± 42.6	7.4 ± 3.4	ns
IFN-γ	ng/L	44.8 ± 39.8	27.9 ± 44.4	17.7 ± 30.9	ns
IL-8	ng/L	97.6 ± 88.4	71.2 ± 83.1	22.1 ± 43.3	ns
IL-6	ng/L	229.3 ± 188.5	136.0 ± 123.1	24.5 ± 12.7	ns
TNF-α	ng/L	155.0 ± 261.1	128.8 ± 215.2	82.1 ± 54.7	ns
IL-1β/IL-10	Ratio	0.40 ± 0.54	0.96 ± 0.81	2.51 ± 0.77	0.034
IFN-γ/IL-10	Ratio	0.61 ± 0.68	1.30 ± 1.21	3.85 ± 3.29	0.015
IL-8/IL-10	Ratio	1.25 ± 1.32	3.71 ± 3.68	4.45 ± 5.10	< 0.001
IL-6/IL-10	Ratio	3.06 ± 2.39	8.25 ± 11.60	9.47 ± 6.38	0.120
TNF-α/IL-10	Ratio	1.87 ± 2.63	8.00 ± 8.63	30.30 ± 21.30	< 0.001

Values were presented as mean ± SD, or % (n).

∗ *P* value: non-survival group versus survival group. ABSI score indicates abbreviated burn severity index score; CRP, C-reactive protein; IFN-γ, interferon γ; ns, no significance; PAI-1, plasminogen activator inhibitor-1; PF-4, platelet factor 4; SOFA, sequential organ failure assessment; sP-selectin, soluble P-selectin; suPAR, soluble urokinase-type plasminogen activator receptor; TBSA, total burn surface area; uPA, urokinase-type plasminogen activator.

### Laboratory parameters and biomarker profiles

Compared with the survival group, the non-survival group had a higher degree of burn injury (higher TBSA, percentage of full-thickness burns, and ABSI score), organ dysfunction (higher SOFA score), and fibrinolytic activity (higher D-dimer; lower suPAR). The non-survival group had higher neutrophils and lower platelets. The platelet activation (lower PF-4) of the non-survival group was significantly lower than that of the survival group. The CRP levels in the non-survival group were lower than those in the survival group, while the IL-10 levels were significantly higher. Compared with the survival group, the imbalance of pro/anti-inflammatory cytokines (IL-1β/IL-10, IFN-γ/IL-10, IL-8/IL-10 and TNF-α/IL-10) in the non-survival group was more significant (Table 1). Compared with the control group, the circulating suPAR levels in the survival group (*P* < 0.001) and non-survival group (*P* = 0.017) were higher.

### Correlations between suPAR and continuous biomarkers

**Fig. 2:**
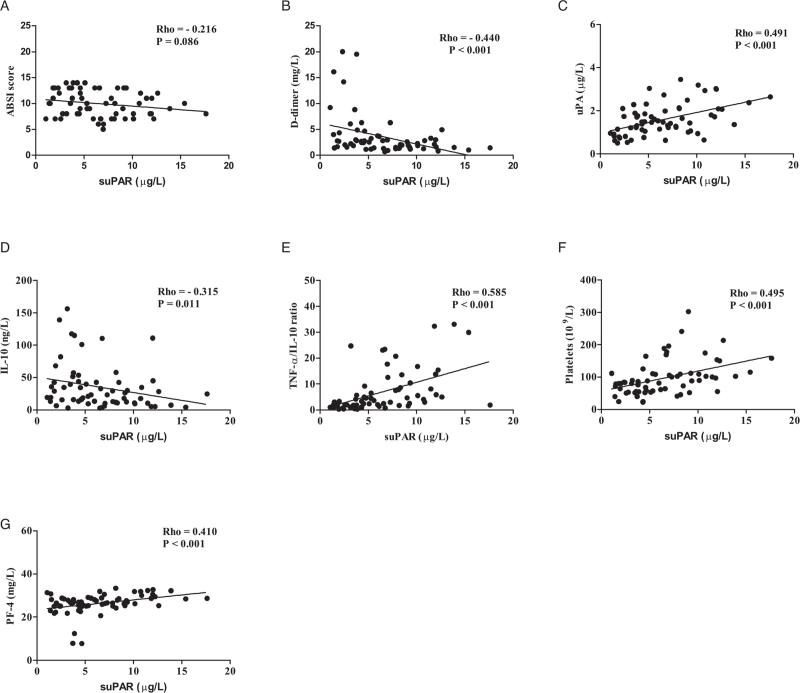
Rho and *P* values are shown for correlations between suPAR (μg/L) and each of the investigated variables in severe burn patients: (A) suPAR versus ABSI score (Score); (B) suPAR versus D-dimer (mg/L); (C) suPAR versus uPA (μg/L); (D) suPAR versus IL-10 (ng/L); (E) suPAR versus TNF-α/IL-10 (Ratio); (F) suPAR versus platelets (109/L); and (G) suPAR versus PF-4 (mg/L). ABSI score indicates abbreviated burn severity index score; PF-4, platelet factor 4; suPAR, soluble urokinase-type plasminogen activator receptor; uPA, urokinase-type plasminogen activator.

The circulating suPAR levels correlated significantly with fibrinolytic activity (D-dimer [Fig. 2B], [rho = −0.440, *P* < 0.001; uPA [Fig. 2C], [rho = 0.491, *P* < 0.001]), pro/anti-inflammatory cytokines (IL-10 [Fig. 2D], [rho = −0.315, *P* = 0.011]; TNF-α/IL-10 ratio [Fig. 2E], [rho = 0.585, *P* < 0.001]), and platelet activation (platelets [Fig. 2F], [rho = 0.495, *P* < 0.001]; PF-4 [Fig. 2G], [rho = 0.410, *P* < 0.001]). No significant correlation was found between the circulating suPAR levels and burn severity (suPAR vs. ABSI score [Fig. 2A], [rho = −0.216, *P* = 0.086]).

### Univariate and multivariate linear regression analyses

We performed linear regression analyses to predict the variables independently associated with suPAR. Univariate regression analysis showed that the variables significantly associated with suPAR were the ABSI score and the levels of platelets, PF-4, D-dimer, uPA, IL-10, IL-1β/IL-10, IL-8/IL-10, and TNF-α/IL-10. When these variables were included in the multivariate linear regression model (the independent variables with *P* values ≤ 0.1 were added to the multivariate regression analysis models to avoid losing potential independent variables), the variables still independently associated with a lower suPAR level were lower PF-4, uPA, and TNF-α/IL-10 levels, as well as a higher D-dimer level (Table 2).

**Table 2:** Linear regression analysis of variables associated with suPAR (μg/L) in server burn patients

	Univariate				Multivariate (R^2^ = 0.611)		
	Unit	β (SE)	*t* value	*P* value	β (SE)	*t* value	*P* value
ABSI score	Score	–0.210 (0.188)	–1.687	0.097	0.160 (0.180)	1.347	0.184
Platelets	10^9^/L	0.433 (0.008)	3.786	< 0.001	0.189 (0.008)	1.763	0.084
PF-4	mg/L	0.359 (0.094)	3.031	0.004	0.258 (0.086)	2.384	**0.021**
D-dimer	mg/L	–0.383 (0.110)	–3.261	0.002	–0.303 (0.093)	–3.067	**0.003**
uPA	μg/L	0.492 (0.567)	4.453	< 0.001	0.299 (0.512)	3.043	**0.004**
IL-10	ng/L	–0.260 (0.013)	–2.122	0.038	0.111 (0.013)	0.941	0.351
IL-6/IL-10	Ratio	0.230 (0.044)	1.861	0.067	0.142 (0.033)	1.526	0.133
IL-8/IL-10	Ratio	0.230 (0.134)	1.861	0.067	0.144 (0.123)	1.269	0.210
TNF-α/IL-10	Ratio	0.488 (0.051)	4.397	< 0.001	0.416 (0.053)	3.646	**0.001**
IL-1β/IL-10	Ratio	0.142 (0.601)	1.133	0.262	–0.231 (0.583)	–1.891	0.064

*P* values were shown in bold for variables with *P* < 0.05 in multivariate analysis. ABSI indicates abbreviated burn severity index; IFN-γ, interferon γ; PF-4, platelet factor 4; SE, standard error; suPAR, soluble urokinase-type plasminogen activator receptor; uPA, urokinase-type plasminogen activator.

### Univariate and multivariate logistic regression analyses of the prognosis

We performed logistic regression analyses to screen variables that could predict the 30-day mortality in severe burn patients. In univariate analysis, the suPAR level, ABSI score, presence of inhalation injury, and lactate level were significantly associated with the 30-day mortality. After adjustment by the multivariate model (age, ABSI score, presence of inhalation injury, and lactate), the suPAR level still correlated negatively with the 30-day mortality (*P* = 0.033) (Table 3). Low circulating suPAR levels (1 μg/L) (odds ratio [OR]: 1.721; [95% CI: 1.045–2.836]; *P* = 0.033) were an independent predictor of an increased 30-day mortality in patients with severe burn (Table 3).

**Table 3:** Univariate and multivariate logistic regression analysis of risk factors associated with 30-day mortality

		Univariate			Multivariate	
Variables	Unit	OR (95% CI)	*P* value	Estimate	OR (95% CI)	*P*-value
suPAR	μg/L	1.584 (1.127–2.225)	**0.008**	0.543	1.721 (1.045–2.836)	**0.033**
Age	Years	0.966 (0.903–1.034)	0.323	–0.027	0.974 (0.879–1.079)	0.612
ABSI score	Score	0.495 (0.317–0.775)	**0.002**	–0.428	0.652 (0.342–1.241)	0.193
Presence of inhalation injury	10^9^/L	0.058 (0.011–0.309)	**0.001**	–1.689	0.185 (0.016–2.137)	0.176
Lactate	mmol/L	0.713 (0.517–0.984)	**0.039**	–0.215	0.806 (0.476–1.368)	0.425

*P* values were shown in bold for variables with *P* < 0.05. ABSI indicates abbreviated burn severity index; CI, confidence intervals; OR, odd ratio; suPAR, soluble urokinase-type plasminogen activator receptor.

### ROC analysis of the suPAR level and classic clinical prognostic biomarkers

**Fig. 3:**
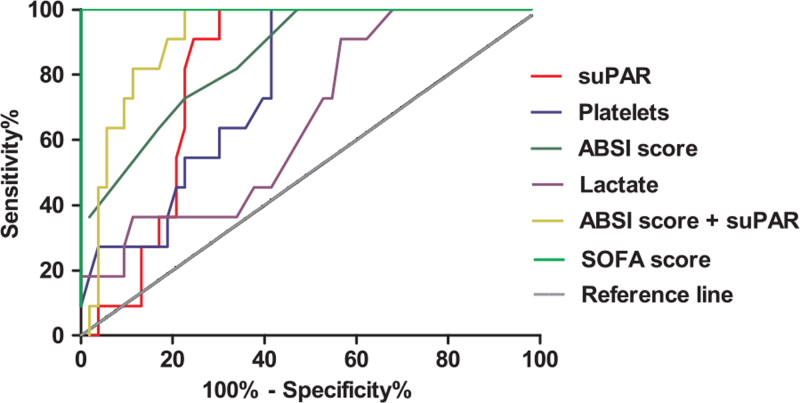
ROC analysis for various prognostic biomarkers to predict the 30-day mortality in patients with severe burn. ABSI score indicates abbreviated burn severity index score; SOFA score, sequential organ failure assessment score; suPAR, soluble urokinase-type plasminogen activator receptor.

To evaluate the value of suPAR in predicting 30-day mortality in severe burn patients, ROC analysis was performed to compare suPAR with classic clinical prognostic biomarkers (platelets, lactate, ABSI score, and SOFA score) (Fig. 3 and Table 4). The ROC-AUC of suPAR was 0.810 (95% CI: 0.708–0.911), which was greater than that of platelets (AUC: 0.766) and lactate (AUC: 0.660) but lower than that of the ABSI score (AUC: 0.853) and SOFA score (AUC: 1.000). The Youden index revealed an optimal cutoff at a suPAR level of 4.70 μg/L. At this cutoff, the sensitivity was 69.8%, and the specificity was 100% (Table 4). When the suPAR was added to the ABSI score, the specificity of predicting 30-day mortality of severe burns increased by 24.6% (Table 4).

**Table 4:** ROC analysis of various prognostic biomarkers for predicting 30-day mortality

	AUC	95% CI	Cut-off values	Sensitivity%	Specificity%
suPAR	0.810	0.708–0.911	4.70 μg/L	69.8	100
Lactate	0.660	0.498–0.822	4.05 mmol/L	90.9	43.4
ABSI score	0.853	0.746–0.960	9.5 score	100	52.8
SOFA score	1.000	1.000–1.000	7.5 score	100	100
Platelets	0.766	0.640–0.891	87 10^9^/L	58.5	100
ABSI score + suPAR	0.919	0.850–0.987	0.100	100	77.4

ABSI indicates abbreviated burn severity index; AUC, area under the curve; ROC, receiver operating characteristic curve; SOFA, sequential organ failure assessment; suPAR, soluble urokinase-type plasminogen activator receptor.

## DISCUSSION

To our knowledge, this study is the first to show that circulating suPAR levels are significantly lower in non-survivors than in survivors at 48 h post-burn. Low suPAR levels early after severe burn may reflect decreased TNF-α/IL-10 ratio and increased hyperfibrinolysis. Circulating suPAR levels at 48 h post-burn are a good prognostic biomarker for patients with severe burn.

Most studies have shown that circulating suPAR levels in non-survivors are significantly higher than those in survivors of critical illness (15, 16, 19, 20). Interestingly, our data revealed that the circulating suPAR levels at 48 h post-burn in non-survivors were significantly lower than those in survivors. The following reasons may explain the contradictory results. First, a recent study (21) showed that immature myeloid cells of Gr-1+ bone marrow are the pathological cause of the increase in suPAR levels. Burn injury can induce immature myeloid cells proliferation in bone marrow (22). However, excessive burn injury may lead to myelosuppression (23). In this study, the burn severity of non-survivors (higher ABSI score) was more severe than that of survivors. This finding may contribute to the decreased circulating suPAR levels in non-survivors. Second, studies (24, 25) showed that activated platelets and their released products may significantly induce uPAR expression on the endothelial surface. Megakaryocytes also express uPAR (26). *In vitro*, activated platelets in patients with paroxysmal nocturnal hemoglobinuria release suPAR, suggesting that activated platelets may be the source of plasma suPAR (27). In the present study, the numbers of platelets and their released products (PF-4) in non-survivors were significantly lower than those in survivors, likely leading to the low expression of uPAR on the endothelial surface and decreased of the circulating suPAR levels in non-survivors. This may be one of the reasons why the circulating suPAR levels of non-survivors were lower than that of survivors in our study. Third, the present study was different from other studies in obtaining suPAR values. In most other studies, suPAR values were obtained at different times in the course of disease, with the maximum value as the final analysis data. In our study, the suPAR values were obtained at 48 h post-burn. Different methods of obtaining suPAR values were likely to lead to such contradictory results. Further animal experiments and external experiments are needed.

The uPA-uPAR system is considered the primary molecule that mediates the extra-fibrinolytic activation pathway (8, 28). uPAR has no catalytic effect but acts to localize plasminogen and uPA to the cell surface, increasing the local reactant concentration (29). Kinetic studies (30) have shown that the catalytic efficiency of uPA bound with uPAR is significantly higher than that of soluble uPA. Under the stimulation of inflammation, uPAR falls off of the cell surface to form suPAR under the action of various proteases (12). suPAR partially or significantly inhibits the binding of uPA to uPAR (27, 31). In this case, suPAR acts as a competitive soluble receptor, thus weakening the role of uPA as a cell surface-associated plasminogen activator (31). In the soluble system containing pro-uPA and plasminogen, the progress of activation is attenuated by suPAR (32). Research has shown that full-length suPAR scavenges uPAR-uPA (33). suPAR can be used as an effective molecular scavenger of uPA in human prostate cancer cells with high uPA-uPAR expression (34), and an increase in suPAR is related to plasminogen inhibition in patients with paroxysmal nocturnal hemoglobinuria (27). Furthermore, D-dimer levels can represent the degree of secondary hyperfibrinolysis (35). Supporting the above literature, D-dimer strongly independently correlated negatively with suPAR by linear regression analysis in this study, even after adjusting for potential confounding factors. In summary, low circulating suPAR levels may reflect secondary hyperfibrinolysis.

In the present study, a strong positive correlation was found between suPAR and TNF-α/IL-10 ratio (*P* < 0.001). Furthermore, in multivariate regression analysis, even after adjusting for potential confounding factors, low suPAR was independently associated with a low TNF-α/IL-10 ratio. The specific mechanism remains unclear. Activated macrophages are polarized to two groups: classical activated macrophages (M1) and selective activated macrophages (M2) (36). M1 macrophages characteristically secrete a large amount of pro-inflammatory cytokine TNF-α, triggering the body's inflammatory response and activating the body's immunity; M2 macrophages characteristically secrete a large amount of anti-inflammatory cytokine IL-10, inhibiting the body's immune response (37, 38). The TNF-α/IL-10 ratio may reflect the degree of macrophage polarization (pro-inflammatory M1/anti-inflammatory M2) in the body (39). uPAR is chemotactic to macrophages (40) and is the regulator of macrophages to absorb apoptotic neutrophils (foam) (41). Macrophages also contribute to uPAR expression in tumor cells *in vivo*(42). uPA-uPAR activates the PI3K-Akt signalling pathway initiating the inflammatory response of macrophages (43). Loss of uPAR leads to increased production of inflammatory cytokines in macrophages, characterized by M1 polarization and impaired phagocytosis (44). uPAR induction of M2 macrophage phenotype expression in the tumor microenvironment may be an important mechanism of uPAR promoting tumor progression (45). uPAR controls macrophage phagocytosis in intestinal inflammation by inducing M2 macrophage polarization (44). Additionally, as a competitive receptor of uPAR, suPAR may weaken the pathophysiological effect of uPAR (27, 31). In summary, low suPAR levels may reflect increased macrophage polarization (from M1 toward M2), likely explaining our experimental results that suPAR independently correlates positively with the TNF-α/IL-10 ratio. Further cell and animal experiments as well as external experiments are needed for verification.

In conclusion, low circulating suPAR levels at 48 h post-burn may reflect decreased TNF-α/IL-10 ratio and increased hyperfibrinolysis in severe burn patients. Many studies have shown that hyperfibrinolysis (1, 4, 46) and the pro/anti-inflammatory imbalance (2, 3, 47, 48) can both lead to serious adverse outcomes after severe burns. This evidence supported our conclusion that low circulating suPAR levels are independently associated with an increased 30-day mortality in patients with severe burn. Further ROC analysis showed that the ROC-AUC of suPAR in predicting the 30-day mortality is higher than that of classic clinical prognostic biomarkers (platelets and lactate). Additionally, suPAR levels showed high stability in the storage state, indicating that the serum suPAR levels were not affected after repeated freezing and dissolution, the diurnal concentration change trend was not obvious, the detection did not depend on fasting or sample collection time, and the detection was simple and inexpensive (49). In conclusion, the prospect of the suPAR level as a prognostic marker of severe burns is promising. The suPAR level is especially suitable for early triage and shunting of large-scale severe burn patients in wartime.

## CONCLUSION

Low circulating suPAR levels at 48 h post-burn in severe burn patients may reflect decreased TNF-α/IL-10 ratio and increased hyperfibrinolysis. suPAR can predict the 30-day mortality in patients with severe burn.

### Limitations of this study

The present study has several limitations. This was an observational study, and its inherent limitations did not allow independent evaluation of causality. Additionally, this study occurred at a single center; thus, extrapolation of its results may be limited. Finally, the sample size of the present study was small, particularly for non-survivors, and the results must be verified by external experiments using larger samples.
